# Reconstructing the course of the COVID-19 epidemic over 2020 for US states and counties: Results of a Bayesian evidence synthesis model

**DOI:** 10.1371/journal.pcbi.1010465

**Published:** 2022-08-30

**Authors:** Melanie H. Chitwood, Marcus Russi, Kenneth Gunasekera, Joshua Havumaki, Fayette Klaassen, Virginia E. Pitzer, Joshua A. Salomon, Nicole A. Swartwood, Joshua L. Warren, Daniel M. Weinberger, Ted Cohen, Nicolas A. Menzies

**Affiliations:** 1 Department of Epidemiology of Microbial Diseases and Public Health Modeling Unit, Yale School of Public Health, Yale University, New Haven, Connecticut United States of America; 2 Department of Global Health and Population, Harvard T.H. Chan School of Public Health, Harvard University, Boston, Massachusetts United States of America; 3 Department of Health Policy, Stanford University, Stanford, California United States of America; 4 Department of Biostatistics and Public Health Modeling Unit, Yale School of Public Health, Yale University, New Haven, Connecticut United States of America; University of Washington, UNITED STATES

## Abstract

Reported COVID-19 cases and deaths provide a delayed and incomplete picture of SARS-CoV-2 infections in the United States (US). Accurate estimates of both the timing and magnitude of infections are needed to characterize viral transmission dynamics and better understand COVID-19 disease burden. We estimated time trends in SARS-CoV-2 transmission and other COVID-19 outcomes for every county in the US, from the first reported COVID-19 case in January 13, 2020 through January 1, 2021. To do so we employed a Bayesian modeling approach that explicitly accounts for reporting delays and variation in case ascertainment, and generates daily estimates of incident SARS-CoV-2 infections on the basis of reported COVID-19 cases and deaths. The model is freely available as the *covidestim* R package. Nationally, we estimated there had been 49 million symptomatic COVID-19 cases and 404,214 COVID-19 deaths by the end of 2020, and that 28% of the US population had been infected. There was county-level variability in the timing and magnitude of incidence, with local epidemiological trends differing substantially from state or regional averages, leading to large differences in the estimated proportion of the population infected by the end of 2020. Our estimates of true COVID-19 related deaths are consistent with independent estimates of excess mortality, and our estimated trends in cumulative incidence of SARS-CoV-2 infection are consistent with trends in seroprevalence estimates from available antibody testing studies. Reconstructing the underlying incidence of SARS-CoV-2 infections across US counties allows for a more granular understanding of disease trends and the potential impact of epidemiological drivers.

## Author summary

Because many COVID-19 infections go undetected, reported numbers of COVID-19 cases and deaths underestimate the true size of the epidemic. To address this problem, we built a model to estimate the number of new SARS-CoV-2 infections over time in each U.S. state and county. In this paper, we present time trends of infections and other disease outcomes from the first reported case in the U.S. until January 1, 2021, for each state and county. The time series of infection estimates suggest that the US epidemic is best described a series of related epidemics, varying in their timing and intensity. We estimate that over a quarter of the US population was infected with SARS-CoV-2 in 2020 and by the end of 2020 0.12% of the population had died from COVID-19. State-level results were consistent with external measures of disease burden, including estimates of SARS-CoV-2 seroprevalence and excess mortality. Our findings help better understand the epidemic in the pre-vaccine era and demonstrate the feasibility of estimating SARS-CoV-2 infections at local levels using routinely reported case and death data.

## Introduction

The numbers of newly diagnosed cases and confirmed COVID-19 deaths are the most easily observed measures of the health burden associated with COVID-19 and have been widely used to track the trajectory of the epidemic at the national, state, and local level.[1, 2] However, there are at least three limitations of using reported cases and deaths for this purpose. First, testing is primarily organized to identify symptomatic individuals, but a large fraction of SARS-CoV-2 infections are asymptomatic, [3] leading to case counts that are substantially smaller than the true incidence of infection. Second, the degree to which case counts undercount infections is sensitive to the availability and utilization of diagnostic testing, which has varied over time and geography.[4–6] For this reason, it can be difficult to distinguish true trends from changes in testing practices. Third, case and death counts are lagging indicators of the transmission dynamics of the pathogen, as they are affected by delays associated with the incubation period, care-seeking behavior of symptomatic individuals, diagnostic processing times, and reporting practices. Taken together, these limitations present challenges to analyses that rely on these metrics as primary signals of SARS-CoV-2 spread.

A better indicator of changes in local transmission is the effective reproduction number (*R*_*t*_), which represents the average number of secondary infections caused by an individual infected at some time *t*.[7] *R*_*t*_ can signal short-term changes in transmission in response to policy and behavioral changes. However, *R*_*t*_ is not a directly observable quantity and estimates of *R*_*t*_ based on raw case reports become biased when reporting delays are incorrectly estimated, [5] weakening their usefulness as a measure of transmission.

Unbiased estimates of COVID-19 cases and the *R*_*t*_ of SARS-CoV-2 can provide more accurate insight into the size and scope of the United States (US) epidemic and inform current and future COVID-19 control policies. A number of modeling approaches have been developed to reconstruct the time series of infections and deaths over the course of the US epidemic. These approaches typically do not allow for variability in case ascertainment and infection fatality ratios (IFRs) across space and time, nor do they attempt to model SARS-CoV-2 infections or COVID-19 deaths at fine spatial scales, such as at the county level.[8, 9]

Here, we present detailed estimates of viral dynamics for all US states and counties, based on a Bayesian statistical model that combines multiple data sources to estimate SARS-CoV-2 infection patterns from observed case notifications and death reports. We apply our model to publicly available COVID-19 case and death data and report on the trajectory of the epidemic from the first reported case (January 13, 2020) until January 1, 2021. The model is available on GitHub (https://github.com/covidestim/covidestim/) as a package for the R programming language (*covidestim*).

## Results

### Analytic overview

**Fig 1:**
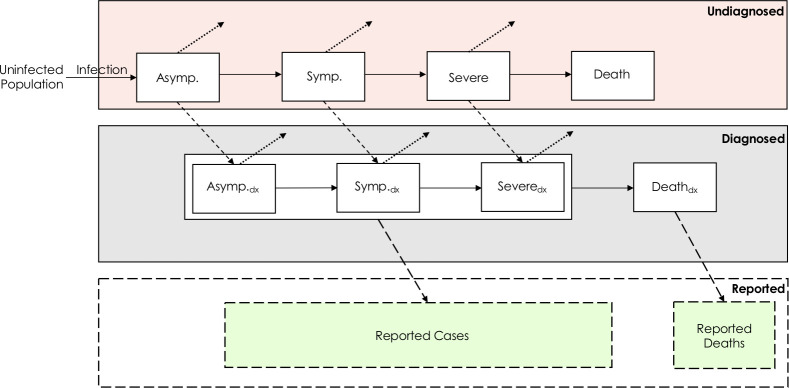
A model schematic of the main health states: *Asymptomatic* (denoted “Asymp.”), *Symptomatic* (denoted “Symp.”), *Severe*, and *Death*. The subscript “dx” indicates that individuals in that state have received a diagnosis of COVID-19. Each transition (denoted with an arrow) has an associated probability and delay distribution. Solid arrows denote disease progression; dotted arrows denote recovery; short dashed arrows denote diagnosis; long dashed arrows denote reporting. All diagnosed cases and deaths are assumed to be reported after a given delay.

We developed a mechanistic model to back-calculate SARS-CoV-2 infections and subsequent outcomes based on reported COVID-19 cases and deaths. In this model the natural history of COVID-19 is represented using four health states: asymptomatic or pre-symptomatic SARS-CoV-2 infection (*Asymptomatic*), symptomatic but not severe COVID-19 disease (*Symptomatic*), severe COVID-19 disease (*Severe*), and death from COVID-19 (*Death*). In each health state (except *Death*) individuals either recover or transition to a more severe state after some delay. Infected individuals can be diagnosed in the *Asymptomatic*, *Symptomatic*, or *Severe* states, and we assume all diagnosed cases and all deaths among diagnosed individuals are reported after a short delay. Fig 1 shows modeled health states and transitions. The model generates several outcomes of epidemiological importance, including *R*_*t*_, total infections, symptomatic cases, total deaths, and case ascertainment; we estimated these outcomes for each US state and county from the start of the epidemic until January 1, 2021.

### Main findings

#### Incidence and R_t_

**Fig 2:**
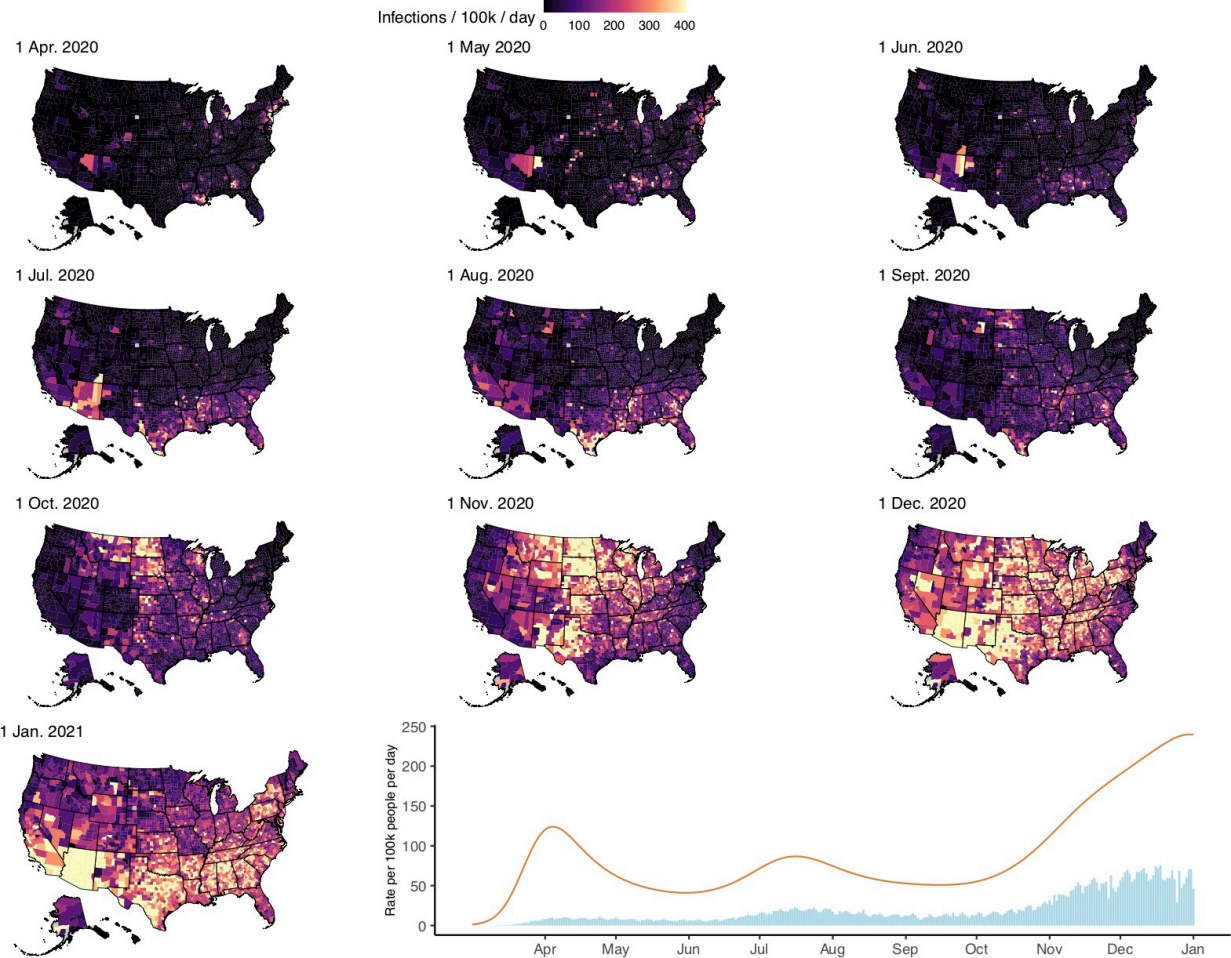
Panels 1–10: County-level infections per 100,000 population per day at 10 timepoints between April 1, 2020 and January 1, 2021. Panel 11: Time series of national SARS-CoV-2 infection estimates (orange line) and reported COVID-19 diagnoses (blue bars) per 100,000 people per day from March 1, 2020 to January 1, 2021. Maps generated using shapefiles from the *alberusa* package for the R programming language: https://github.com/hrbrmstr/albersusa.

**Fig 3:**
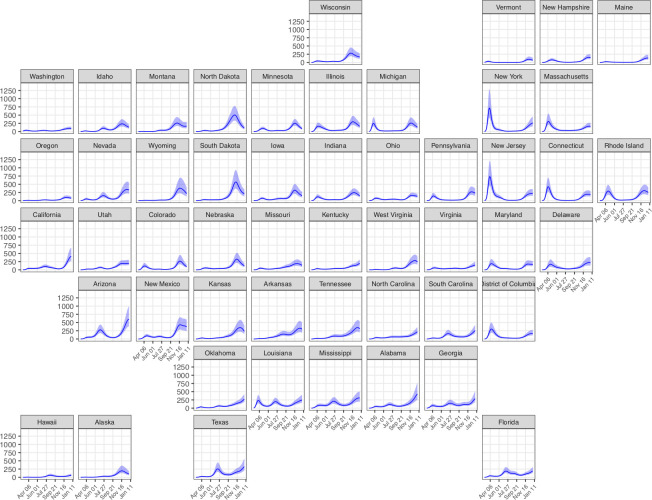
Incident infections per 100,000 residents per day for each US state from March 1, 2020 to January 1, 2021. Shaded areas represent 95% credible intervals.

The SARS-CoV-2 epidemic in the US consisted of a series of related outbreaks, which varied greatly in both the intensity of transmission and the extent of geographic spread (Fig 2). The March outbreak in New Jersey was the largest per population in a single state; on April 4, we estimate that New Jersey had 732 (95% credible interval: 464, 1206) infections per 100,000, and 16% (10%, 26%) of all infections in the US on that day. Local surges in infections during the fall and winter of 2020 rivaled New Jersey and New York’s spring outbreaks in scale, but occurred in the context of a more generalized US epidemic. South Dakota, for example, had its highest per capita infections of 2020 on November 8 (569 [365, 940] infections per 100,000), but accounted for just 1.2% (0.7%, 1.9%) of all US infections that day. Forty-five states experienced the highest daily infections per capita in November or December (Fig 3). Model fit to data can be found in S1 Fig.

While most states and counties had lower levels of transmission during the summer months, few achieved established thresholds of low levels of community transmission, defined as fewer than 20 confirmed cases per 100,000 per week. [10] We estimate that only four states (Alaska, Montana, Vermont, and West Virginia) had fewer than 20 symptomatic cases per 100,000 inhabitants per week after transmission was established locally. Notably, Vermont remained below this threshold from the week of May 11 until the week of September 28.

**Fig 4:**
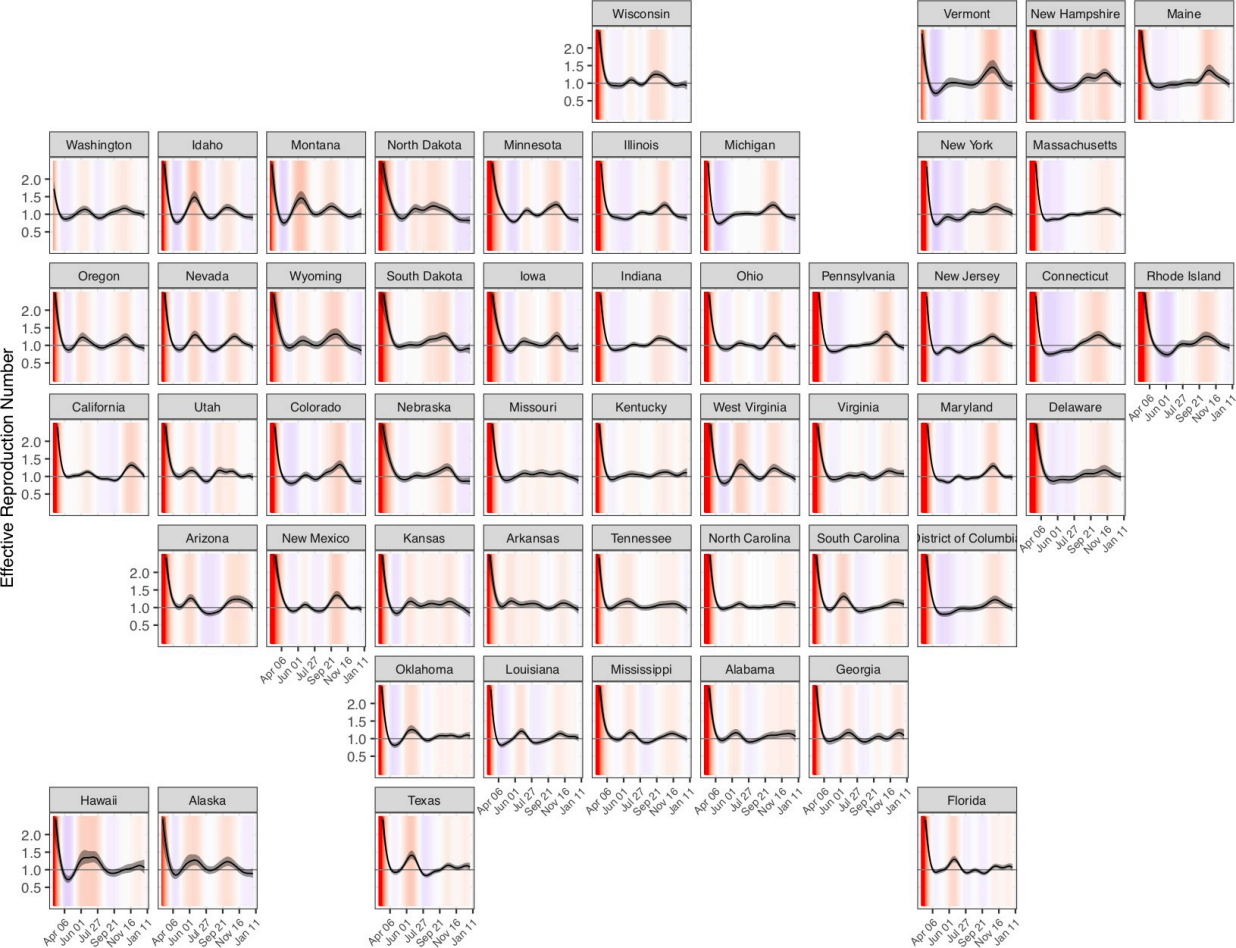
*R*_*t*_ estimates for each US state from March 1, 2020 to January 1, 2021. Background colors indicate whether *R*_*t*_ is substantially greater than 1 (red), close to 1 (white), or substantially less than 1 (blue). Grey line indicates *R*_*t*_ = 1. Shaded areas represent 95% credible intervals.

Estimates of *R*_*t*_ at the start of the epidemic varied greatly by state. The median state-level estimate of *R*_*t*_ on the first day a case was reported in each state was 3.4 (range: 1.7 [1.5–2.0] in Washington to 5.9 [4.3–8.2] in New York). Throughout April, *R*_*t*_ estimates dropped substantially. Over the period May 1, 2020 to January 1, 2021, state-level estimates of *R*_*t*_ ranged from 0.7 (0.6, 0.8) to 1.5 (1.3, 1.7) (Fig 4).

#### Percent Ever-Infected with SARS-CoV-2

**Fig 5:**
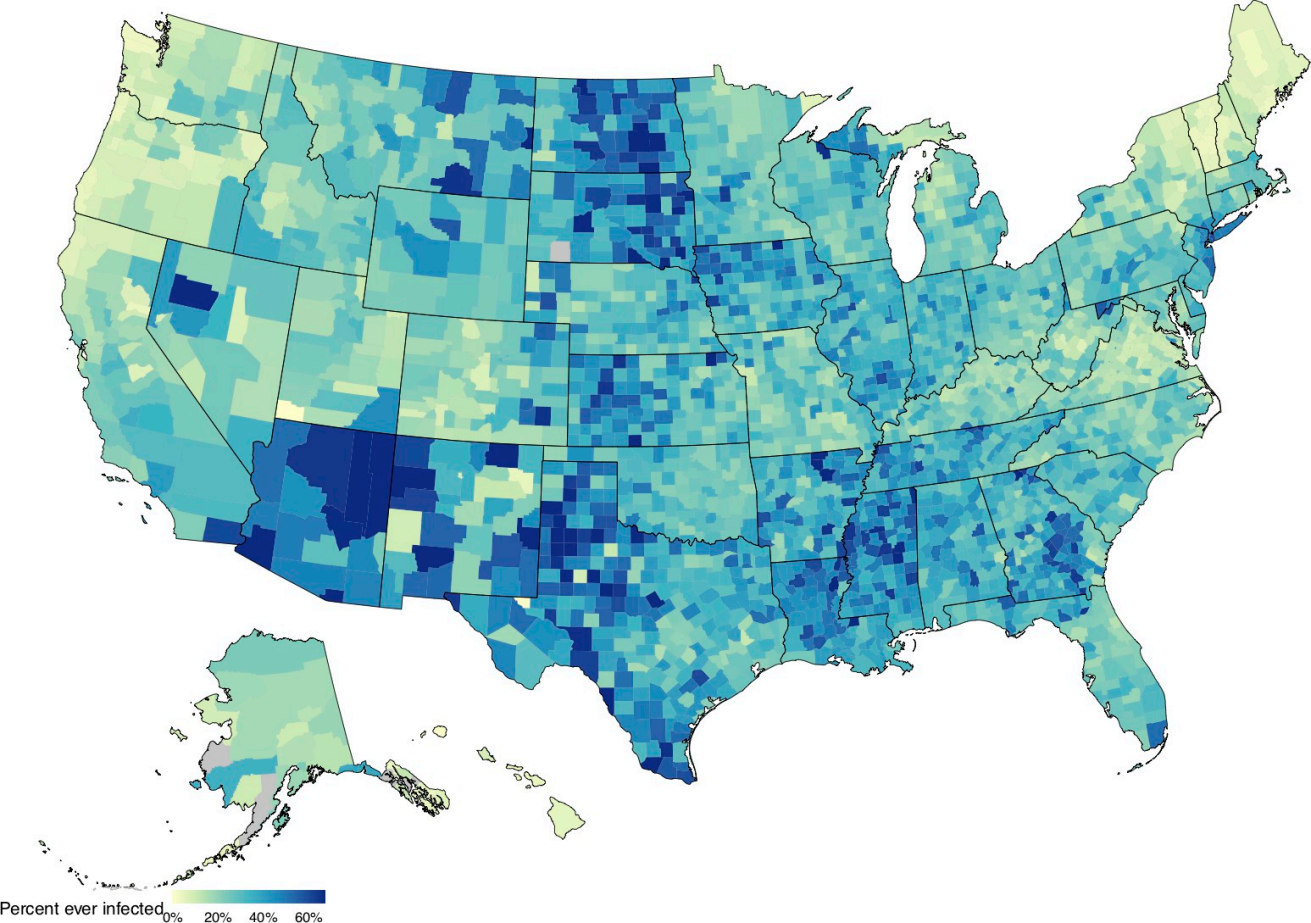
Percentage of the population ever-infected with SARS-CoV-2 as of January 1, 2021. Map generated using shapefiles from the *alberusa* package for the R programming language: https://github.com/hrbrmstr/albersusa.

For each county, we calculated the percentage of the population ever-infected as the sum of all estimated infections divided by county population on January 1, 2021 (Fig 5). This cumulative infection estimate is distinct from reported seroprevalence estimates, as seroprevalence measures may be affected by the lower immune response among individuals with mild/asymptomatic infection, possible waning of antibody titers,[11, 12] and non-representativeness of sampled populations.[13] By January 1 2021, we found that the percent of the population ever-infected exceeded 50% in 303 (9.7%) counties and exceeded two-thirds of the population in 42 (1.3%) counties. Conversely, the percent ever-infected was less than 10% in 144 (4.6%) counties and less than 5% in 37 (1.2%) counties. Based on the sum of state estimates (posterior medians), we estimate that 28% of the US population had been infected with SARS-CoV-2 by January 1, 2021. Across states, the percentage ever-infected ranged from 6.5% (4.2%, 11.1%) in Vermont to 45.7% (30.7%, 67.1%) in Arizona (Fig 5).

On January 1, 2021, the US had reported 348,055 cumulative COVID-19 deaths.[14] Based on the sum of state estimates (posterior medians), we estimate there were 404,214 cumulative COVID-19 deaths as of January 1, 2021, 16.1% greater than cumulative reported deaths and approximately 0.12% of the US population on January 1, 2020. Estimates of the size of the infected population were sensitive to assumptions about the IFR, with higher IFR values producing lower estimates of the infected population (S2 Fig). Other major epidemiological outcomes (*R*_*t*_, symptomatic cases, severe cases, COVID-19 deaths) had weak relationships with the IFR (absolute rank correlations all <0.2). Alternative assumptions for how county-level IFRs relate to state-level values had a modest impact on cumulative infection estimates (S4 Fig). Cumulative infection estimates and other epidemiological outcomes showed modest changes under different assumptions regarding the time course of COVID-19 disease progression (S5A–S5C Fig) and reporting delays (S5D and S5E Fig).

#### Infection ascertainment

**Fig 6:**
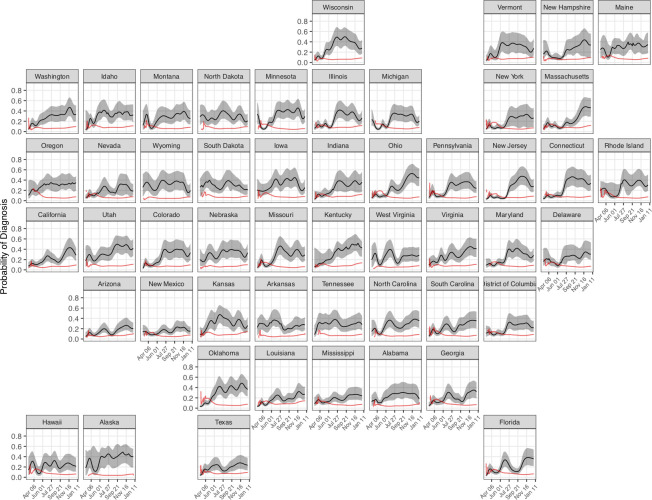
The probability that a person infected with SARS-CoV-2 on a given day will be diagnosed for each US state from March 1, 2020 to January 1, 2021. Shaded areas represent 95% credible intervals. The red line represents a seven-day moving average of the fraction of positive tests.

The probability that an infection is diagnosed changed substantially over the course of the U.S. epidemic. Ascertainment was low in the months of March, April, and May 2020. The national median state-level infection ascertainment (based on state-level posterior medians) in this period was 13.2% (range: 3.2%, 39.7%). Infection ascertainment improved steadily through November 2020, excluding a period of lower ascertainment in July and August; the national mean probability of diagnosis fluctuated between 24% and 35% between September 1, 2020 and January 1, 2021. Infection ascertainment estimates varied significantly across states, and state-level estimates were highly uncertain (Fig 6). Only 3 states achieved greater than 50% ascertainment at any point in 2020 (based on posterior median). State-level model estimates of infection ascertainment each day were negatively correlated with the seven-day moving average fraction of tests that had a positive result[15] (Spearman rank correlation (*ρ*) = -0.36, p < 0.001). From the introduction of SARS-CoV-2 in the US until January 1, 2021, we estimate that 22.4% of infections were identified and reported. Infection ascertainment estimates were sensitive to assumptions about the IFR, with higher IFR values producing higher estimates of the fraction of infections identified and reported (S2 Fig). Ascertainment estimates were also sensitive to the natural history delays (S5A–S5C Fig) and reporting delays (S5D and S5E Fig) assumed in the analysis.

### Comparisons to External Covid-19 Burden indicators

**Fig 7:**
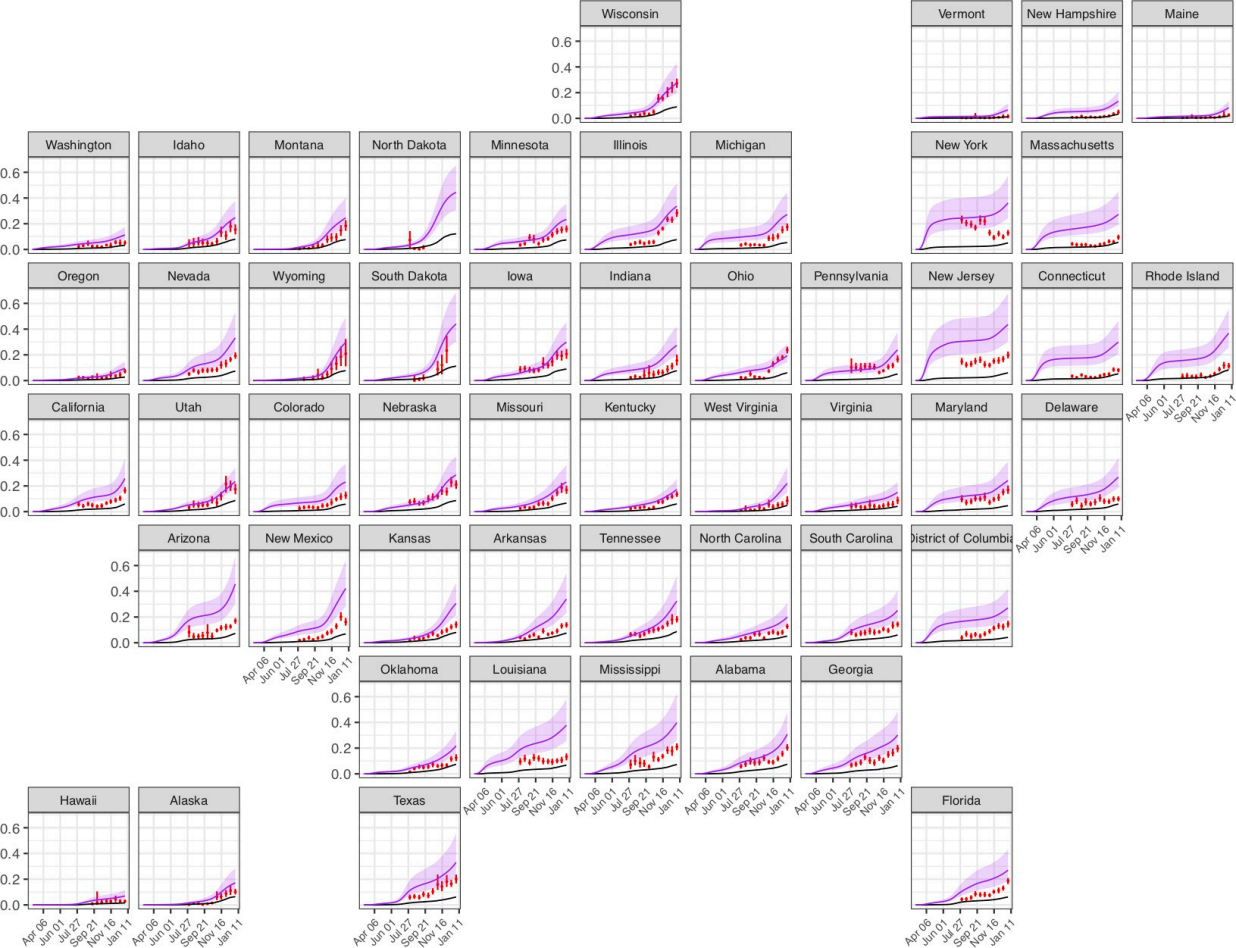
Comparison of the estimated percent ever-infected with SARS-CoV-2 (purple line, shaded areas represent 95% credible intervals) to CDC seroprevalence estimates from commercial laboratory data (red vertical line) and cumulative reported cases (black line) for each US state from March 1, 2020 to January 1, 2021.

We compared our estimates of the percent ever-infected with SARS-CoV-2 to U.S. Centers for Disease Control (CDC) seroprevalence estimates drawn from commercial laboratory data,[16] acknowledging previously noted differences between these outcomes. Derived from a convenience sample of blood specimens collected for reasons unrelated to COVID-19, the seroprevalence estimates provide state-level evidence on SARS-CoV-2 antibody test positivity at multiple time points (Fig 7). However, these estimates are incomplete in some states (e.g. South Dakota), and the series of values declines over time in others (e.g. New York). Comparing these estimates to other reported indicators of cumulative disease burden on December 31, 2020, the modeled estimates of the percent ever-infected were more strongly correlated with cumulative hospitalizations (Spearman rank correlation (ρ) = 0.62) and cumulative reported deaths (ρ = 0.82) than the CDC seroprevalence estimates (ρ = 0.41 and 0.37 for hospitalizations and deaths respectively).

**Fig 8:**
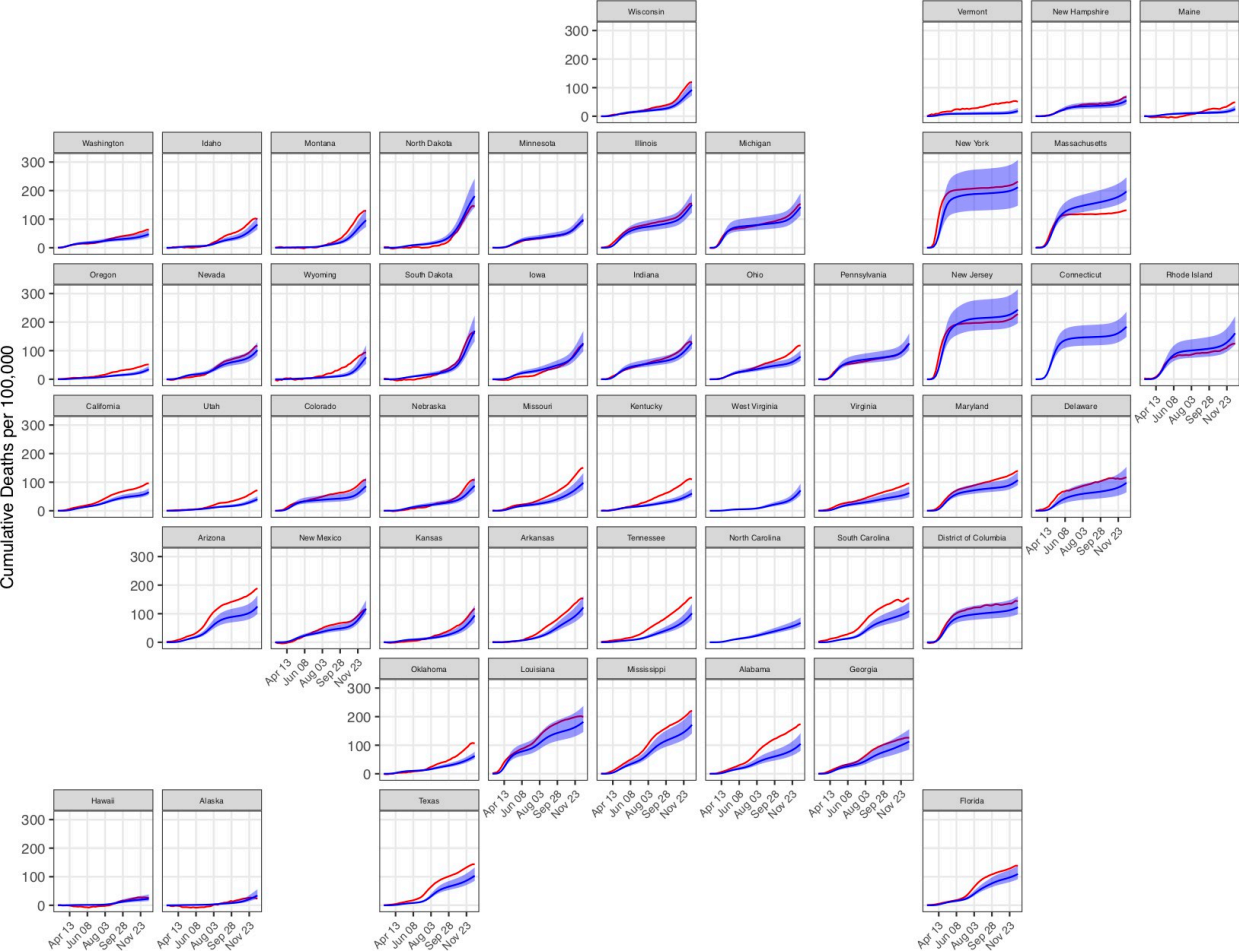
Comparison of cumulative COVID-19 deaths (blue) to cumulative excess all-cause mortality (red) for each US state from March 7 to December 19, 2020. Shaded areas represent 95% credible intervals.

In addition, we compared model estimates of cumulative COVID-19 deaths (detected and undetected) to state-level estimates of excess all-cause mortality, which reflect both COVID-19 deaths and deviations from expected levels and patterns in non-COVID-19 deaths, [6] (Fig 8) at each weekly timepoint from March 7 to December 19, 2020. On average, modeled estimates of cumulative COVID-19 deaths are less than or approximately equal to estimates of excess all-cause mortality. Notably, three states (Alaska, Hawaii, Maine) have extended periods where the estimated all-cause mortality did not exceed all-cause mortality from previous years (i.e. excess mortality was negative); in periods where all-cause mortality is higher than expected, our estimates of COVID-19 deaths correlate strongly with excess mortality estimates (Spearman rank correlation (*ρ*) = 0.95, p < 0.001). Additionally, model estimates of cumulative COVID-19 deaths exceed estimates of excess all-cause mortality in four states (New Jersey, North Dakota, Massachusetts and Rhode Island). Estimates of excess all-cause mortality were not available for Connecticut, North Carolina, or West Virginia.

## Discussion

We present detailed estimates of the dynamics of SARS-CoV-2 infections in US states and counties through the end of 2020. We found that the viral dynamics are best described as a series of related local and regional epidemics, differing in their timing and magnitude even within individual states. This is evident in the large variation in state- and county-level estimates of percent ever-infected as of January 1, 2021. As case ascertainment has also varied over space and time, these estimates provide insights beyond those that can be inferred from cumulative case counts alone. Ascertainment of infection improved markedly after the first months of the US epidemic, but remained low nationally; we conclude that the reported cumulative case count was approximately one-quarter of the true number of US infections at the end of 2020.

Most notably, we found that model estimates of cumulative infections differ from seroprevalence estimates produce by the CDC. Our estimates of cumulative infections are more strongly correlated with cumulative hospitalizations and deaths across states, potentially reflecting biases in the empirical seroprevalence estimates. Seroprevalence studies have a number of known limitations, including the use of non-representative samples [13] and possible reduced sensitivity associated with waning of antibody titers, as has been reported for some tests. [11, 12] A comparison between model estimates and seroprevalence data therefore suggests that this method provides valuable information about the incidence of infection over time.

The Bayesian estimation approach used for this analysis makes a number of simplifying assumptions. To reduce model complexity, we rely on fixed distributions to describe delays in disease progression and detection. Because we anchor the analysis on death data (under the assumption that deaths were more consistently reported than cases over the course of the epidemic), model estimates are sensitive to IFR estimates. These IFR estimates are themselves uncertain, being derived from the comparison of death counts to seroprevalence estimates and therefore inheriting the potential biases of these studies. [11–13] While we allow for variation in IFR values at state- and county-level, this variation is based on proxy measures (differences in the age distribution of COVID-19 deaths, differences in reported prevalence of risk factors for severe disease), which may weaken the robustness of this approach. Moreover, it is likely that the IFR has varied over time within each modeled geography due to changes in the age distribution of infections, yet the limited reporting of age-stratified data means that these age-based changes could not be represented in the model. We also allow for modest under-reporting of COVID-19 deaths, consistent with empirical studies of COVID-19 death reporting, [11, 17, 18] but this is an uncertain input to our analysis. For other modeling assumptions—in particular, the delay distributions quantifying the lag between infection and reporting—we assumed consistent values across all locations, due to a lack of data on how these vary across states and counties. Finally, we assume that a previously infected individual cannot be re-infected with SARS-CoV-2. While waning antibody titers suggest that re-infection is possible over time, we do not believe that our assumptions about re-infection meaningfully impact our results. [19, 20]

In addition, we used data that have been aggregated from state-level reporting mechanisms, which are vulnerable to a number of potential sources of bias. States vary in their reporting criteria (e.g. reporting the number of positive tests as opposed to number of individuals who have tested positive) and the average delay between case detection and reporting. Data are also subject to occasional revisions, often implemented as a single-day change in the cumulative count of cases or deaths. Taken together, these data irregularities lead to additional variance in the reported data and a reduction in the precision of reported estimates. While line-list data would likely improve the precision of model estimate [21], these data are not widely available in the US. Despite these limitations, the method described here may represent an improvement over similar modeling approaches that do not allow for case ascertainment rates and infection fatality ratios that vary over both space and time, [8, 9, 22, 23] or that estimate *R*_*t*_ using model outputs rather than as part of the modeling framework. [9, 21, 23] Furthermore, our approach uses changes in case and death data to estimate changes in transmission, while others approaches make use of more indirect data on mobility [8, 22] or similar proxies [23] to signal changes in transmission. While mobility has a mechanistic relationship with disease transmission, the association between movement data and viral transmission is complex and variable across time and space, possibly because of changes in mask use and other non-pharmaceutical interventions. [24, 25]

In conclusion, the modeling approach described here provides a coherent framework for simultaneously estimating the trend in SARS-CoV-2 infections and the fraction of the population that has been infected previously, providing key information on the viral dynamics at county- and state-levels. While the deployment of effective vaccines against the virus represents a great hope for the control of SARS-CoV-2 transmission, vaccine hesitancy and the emergence of more transmissible variants [26] present an ongoing challenge to disease control in the US. Understanding the course of the epidemic in the pre-vaccine era can help guide decision making in a landscape with heterogenous vaccine coverage. Ongoing, local evidence on trends in *R*_*t*_ and new and cumulative infections will continue to be important for both governments and individuals.

## Methods

We developed a mechanistic model that uses reported case and death data to back-calculate the natural history cascade of SARS-CoV-2. The model estimates the expected number of cases and deaths reported on a given day as the convolution of the time series of diagnosed cases and deaths (among diagnosed individuals) and fixed reporting delay distributions; the expected number of diagnoses on a given day is estimated with health-state specific and time-varying probabilities of diagnosis. The model represents the natural history of COVID-19 as a series of health state transitions with associated probabilities and delays (Fig 1). The model utilizes delay distributions associated with health state progression, time-invariant probabilities of transitioning from *Asymptomatic* to *Symptomatic* and from *Symptomatic* to *Severe*, and a time-varying probability of transitioning from *Severe* to *Death*. The number of individuals entering *Asymptomatic* is a function of the serial interval, the fraction of the population not yet infected, and *R*_*t*_; *R*_*t*_ is modeled using a log-transformed cubic b-spline.

### Data

For every state and county in the United States, we extracted daily data on reported COVID-19 cases and deaths from a repository compiled by the Johns Hopkins Center for Systems Science and Engineering (CSSE) [14]. We calculated the time series of new cases and deaths as the difference between cumulative counts reported on consecutive days. In instances in which the reported cumulative count decreased from one day to the next, we assumed that there were zero new cases or deaths on each day until the cumulative count exceeded the previous maximum. In several instances the data reported by CSSE fail to capture the beginning of the epidemic in early 2020, or exhibit irregularities during this period. To reconstruct the time series for this period we used data compiled by the Covid Tracking Project. [15]

### Mathematical model

We constructed a deterministic mathematical model relating reported cases and deaths to unobserved COVID-19 natural history. A flexible function for *R*_*t*_ determines the number of individuals infected on a given day, and the model then tracks the progression of the infected cohort through health states of increasing disease severity, with modeled quantities—*A*_*t*_ (*Asymptomatic*), *S*_*t*_ (*Symptomatic*), *V*_*t*_ (*Severe*), and *D*_*t*_ (*Death*)—reflecting the number of individuals entering a given health state on day *t*. From each health state, an individual can either recover or progress to the next health state, with this transition governed by a defined delay distribution. Ultimately, the model estimates an expected number of reported cases and deaths on each day, which are fit to observed data via negative binomial likelihood functions.

### New infections

We modeled the daily number of newly-infected individuals (*A*_*t*_) entering the *Asymptomatic* state. For each modeled location, we specified a random intercept (*A*_0_) 28 days before the first reported COVID-19 case, and calculated changes in *A*_*t*_ as a function of the effective reproduction number (*R*_*t*_) and the mean serial interval (*z*), measured in days (derivation shown in supplement).


$$A_{t+1}=A_{t}R_{t}^{\frac{1}{z}}\;for\;t\geq 0$$ (1)

We modeled the time trend in *R*_*t*_ using a log-transformed cubic b-spline (*X*_*R*,*t*_) with knots every 10 days (S3 Fig), allowing flexibility in the evolution of the epidemic curve over time. Penalties on first and second differences of the spline parameters were used to dampen oscillations not supported by the data. We assumed that individuals can only be infected once and multiplied the spline by the fraction of the population (*N*) uninfected at each timepoint, penalizing *R*_*t*_ towards zero as the population ever-infected approaches 100%.


$$R_{t}=X_{R,t}\left(1-\frac{\sum_{i=0}^{t}A_{i}}{N}\right)$$ (2)

### Disease progression

We assumed that a fraction of individuals with asymptomatic disease (*p*_*S*_) progress to the *Symptomatic* state. The delay from infection to symptoms was assumed to follow a Gamma distribution, with *ρ*_*S*,*i*_ representing the fraction progressing between *i* and *i+1* days after infection, among those progressing to the symptomatic state. We tested the sensitivity of model outcomes to the choice of symptom onset delay (S5A Fig).


$$S_{t}=\sum_{i=0}^{t}A_{t-i}p_{S}\rho_{S,i}$$ (3)

Similarly, a fraction of individuals in the *Symptomatic* state (*p*_*V*_) were assumed to progress to the *Severe* state, with Gamma-distributed delay distribution *ρ*_*V*,*i*_. A fraction of individuals with severe disease (*p*_*D*,*t*_) die, with Gamma-distributed delay distribution *ρ*_*D*,*i*_. We tested the sensitivity of model outcomes to the choice of delay to severe disease (S5B Fig) and to death (S5C Fig).


$$V_{t}=\sum_{i=0}^{t}S_{t-i}p_{V}\rho_{V,i}$$ (4)

$$D_{t}=\sum_{i=0}^{t}V_{t-i}p_{D,t}\rho_{D,i}$$ (5)

With the exception of *p*_*D*,*t*_, disease progression parameters were not allowed to vary over time. For *p*_*D*,*t*_ we assumed higher values applied in early 2020, reflecting higher case fatality among individuals with severe disease early in the epidemic due to later presentation and lower effectiveness of treatment at that time. We modeled the time trend in *p*_*D*,*t*_ as the product of *p*_*D*0_ (the progression probability after early 2020) and ${OR}_{p_{D,t}}$, an odds ratio describing the elevated case fatality early in the epidemic (Eq 6). ${OR}_{p_{D,t}}$ was operationalized using a declining sigmoid curve (1.0 minus the Normal cumulative distribution function Φ) with an inflection point on May 1 2020 (Eq 7). In this equation, *μ* is equal to the number of days between the start of the model (*t* = 0) and May 1^st^ 2020, *σ* is equal to 21 days, and $a_{p_{D}}$ represents an uncertain parameter for the *additional* mortality risk early in the epidemic. This formulation ensures that ${OR}_{p_{D,t}}$ asymptotes toward 1.0 as *t* increases after May 1^st^ 2020.


$$\frac{p_{D,t}}{1-p_{D,t}}=\frac{p_{D,0}}{1-p_{D,0}}{OR}_{p_{D,t}}$$ (6)

$${OR}_{p_{D,t}}=1+\left(1-\Phi (\frac{t-\mu }{\sigma })\right)a_{p_{D}}$$ (7)

While vaccination would also affect disease progression probabilities, we assumed that vaccination coverage was insufficient to impact disease natural history during the study period.

### Infection fatality ratio

We assumed that the infection fatality ratio (IFR) differs across states and counties, reflecting differences in the age distribution of the epidemic and differences in the prevalence of medical risk factors for severe COVID-19 disease. First, we calculated the age distribution of infections for each state, based on the reported age distribution of COVID-19 deaths [27] and published age-specific IFRs. [28] Second, we used these age distributions to calculate an average IFR for each state, weighting the age-specific IFRs by the fraction of the population in each age group. This produced a national average IFR of 0.35, which we believe to be implausibly low; we rescaled state-level values to produce a national average IFR of 0.5%. [29] As the age-distribution of COVID-19 deaths was not available at the county-level, we estimated county-level IFR values by multiplying the state-average IFR by the prevalence of medical risk factors for severe COVID-19 disease in each county relative to the rest of the state. [30] To test the impact of this assumption, we performed a sensitivity analysis with a simpler approach that holds all county-level IFRs equal to the state level (S4 Fig). To understand the implications uncertainty in the IFR for modeled estimates of the infected population, we plotted the relationship between these two quantities in the fitted model outcomes.

### Diagnosis

We assumed that infected individuals could be diagnosed from the *Asymptomatic*, *Symptomatic*, or *Severe* states, and that diagnosis would not affect disease progression. To reduce model complexity, we assumed that diagnosis in the Asymptomatic state only occurs among individuals who will not progress to the Symptomatic state. The daily number of these diagnoses is denoted ${\hat{A}}_{t}$ (with the ^ used to indicate quantities related to diagnosis). The fraction of these individuals diagnosed (*q*_*A*,*t*_) was assumed to vary over time, to allow for changes in case ascertainment over the course of the epidemic. The delay to diagnosis was defined by ${\hat{\rho }}_{A,i}$, which is described by a Gamma distribution.


$${\hat{A}}_{t}=\sum_{i=0}^{t}A_{t-i}q_{A,t-i}{\hat{\rho }}_{A,i}(1-p_{S})$$ (8)

To estimate the number diagnosed from the *Symptomatic* state (${\hat{S}}_{t}$) we assumed a time-varying probability of diagnosis *q*_*S*,*t*_ and delay to diagnosis ${\hat{\rho }}_{S,i}$.


$${\hat{S}}_{t}=\sum_{i=0}^{t}S_{t-i}q_{S,t-i}{\hat{\rho }}_{S,i}$$ (9)

The number diagnosed from the *Severe* state (${\hat{V}}_{t}$), was calculated based on a time-invariant probability of diagnosis (*q*_*V*_) and delay to diagnosis ${\hat{\rho }}_{V,i}$. These were applied after subtracting individuals developing severe disease who had been previously diagnosed at *Symptomatic* (${\bar{V}}_{t}$).


$${\bar{V}}_{t}=\sum_{i=0}^{t}S_{t-i}q_{S,t-i}p_{V}\rho_{V,i}$$ (10)

$${\hat{V}}_{t}=\sum_{i=0}^{t}\left(V_{t-i}-{\bar{V}}_{t-i}\right)q_{V}{\hat{\rho }}_{V,i}$$ (11)

Time-varying diagnosis probabilities (*q*_*A*,*t*_, *q*_*S*,*t*_) were calculated as a function of *q*_*V*_:

$$q_{S,t}=q_{V}X_{q_{S},t}$$ (12)

$$q_{A,t}=q_{V}X_{q_{S},t}{RR}_{q_{A}}$$ (13)

In Eqs 12 and 13, $X_{q_{S},t}$ is operationalized as a cubic b-spline that has been logit-transformed to fall within the unit interval, with knots spaced 21 days apart, and with penalties on first and second differences of the spline parameters. ${RR}_{q_{A}}$ is constrained to fall in the unit interval, so that that *q*_*A*,*t*_≤*q*_*S*,*t*_≤*q*_*V*_ for all *t*.

### Reporting

We assumed that all diagnosed COVID-19 cases were reported. The number of diagnoses reported on a given day (${\dot{C}}_{t}$, with the ‘·’ used to indicate quantities related to reporting) was calculated as the sum of diagnoses from *Asymptomatic*, *Symptomatic* and *Severe* states, with reporting delay ${\dot{\rho }}_{C,i}$.


$${\dot{C}}_{t}=\sum_{i=0}^{t}\left({\hat{A}}_{t-i}+{\hat{S}}_{t-i}+{\hat{V}}_{t-i}\right){\dot{\rho }}_{C,i}$$ (14)

The reported number of COVID-19 deaths (${\dot{D}}_{t}$) were calculated from the number of diagnosed individuals who subsequently died $({\hat{D}}_{t}).\;{\hat{D}}_{t}$ was calculated as the sum of deaths among individuals diagnosed from the *Symptomatic* and *Severe* states, represented by the first and second terms in Eq 15, respectively. We assumed that all deaths among diagnosed COVID-19 cases were reported, with reporting delay ${\dot{\rho }}_{D,i}$.


$${\hat{D}}_{t}=(\sum_{i=0}^{t}{\bar{V}}_{t-i}p_{D,t}\rho_{D,i})+(\sum_{i=0}^{t}\left(V_{t-i}-{\bar{V}}_{t-i}\right)q_{V}p_{D,t}\rho_{D,i})$$ (15)

$${\dot{D}}_{t}=\sum_{i=0}^{t}{\hat{D}}_{t-i}{\dot{\rho }}_{D,i}$$ (16)

We tested the sensitivity of model outcomes to the choice of reporting delays (S5D and S5E Figs).

### Data likelihood

We specified negative binomial likelihood functions to fit the model to observed cases (*Y*_*C*,*t*_) and death data (*Y*_*D*,*t*_).


$$Y_{C,t}∼NegBin({\dot{C}}_{t},\phi_{C})$$

$$Y_{D,t}∼NegBin({\dot{D}}_{t},\phi_{D})$$

To account for variation in daily reported cases and deaths, we fit the likelihood function using a seven-day moving average of input data. The negative binomial dispersion parameters (*ϕ*_*C*_, *ϕ*_*D*_) were estimated simultaneously, allowing for additional variance in the observed time series.

### Model parameters

Model parameters are shown in Table 1. The distributions of delays from infection to symptom onset, symptom onset to severe disease, and severe disease to death are used in the model as fixed inputs and can be found in Table 2. S6 Fig shows the comparison of prior and posterior distributions for key parameters listed in Table 1.

**Table 1:** Model parameters.

Model Parameter	Mean, std. Deviation	Distribution	Type	Source
Log of New Infections At T = 0 (***A***_**0**_)	0,10	Normal(0,10)	prior	Assumed
***X***_***R*,*t***_ Spline Parameters	0,3	Normal(0,3)	prior	Assumed
First Derivative of ***X***_***R*,*t***_ Spline Parameters	0,0.5	Normal(0,0.5)	prior	Assumed
Second Derivative of ***X***_***R*,*t***_ Spline Parameters	0,0.1	Normal(0,0.1)	prior	Assumed
Serial Interval	5.8, 0.5	Gamma(129.1, 22.25)	prior	[31]
Probability of Developing Symptoms If Infected	0.59, 0.16	Beta(5.14, 3.53)	prior	[3,32–34]
Probability of Becoming Severely Ill If Symptomatic	0.09, 0.06	Beta(1.89, 20.00)	prior	[35,36]
Probability of Death for All Infections (national average)	0.005, 0.001	Beta(15.9, 3167)	prior	[28,29]
Probability of Death for Severe Infections	0.15, 0.03	Beta(28.2, 162.3)	prior	[36]
Additional Risk of Mortality Prior to May 1 2020 ($a_{p_{D}}$)	1.34, 0.39	Gamma(12.03, 8.99)	prior	Assumed
Rate Ratio, Diagnosis at Asymptomatic Vs. Symptomatic	0.1, 0.07	Beta(2,18)	prior	Assumed
Rate Ratio, Diagnosis at Symptomatic Vs. Severe	0.5, 0.22	Beta(2,2)	prior	Assumed
Probability of Diagnosis at Severe	0.72, 0.16	Beta(20,5)	prior	Assumed
Dispersion Parameter for Reported COVID-19 Cases (1/σ)^2^	0.8, 0.6	Half-Normal(0,1)	prior	[37]
Dispersion Parameter for Reported COVID-19 Deaths (1/σ)^2^	0.8, 0.6	Half-Normal(0,1)	prior	[37]
Scaling Factor: Time to Diagnosis Relative to Time in Symptomatic State	0.5, 0.22	Beta(2,2)	prior	Assumed
Scaling Factor: Time to Diagnosis Relative to Time in Severe State	0.5, 0.22	Beta(2,2)	prior	Assumed

**Table 2:** Delay Distributions.

Delay	Mean, std. Deviation	Distribution	Source
Infected to Symptomatic (Days)	5.6, 3.1	Gamma(3.41, 0.61)	[38]
Symptomatic to Severe (Days)	7.5, 5.8	Gamma(1.72, 0.22)	[39]
Severe to Death (Days)	9.1, 6.3	Gamma(2.10, 0.23)	[40]
Case Reporting Delay	2.2, 1.5	Gamma(2.2, 1)	Assumed
Death Reporting Delay	2.2, 1.5	Gamma(2.2, 1)	Assumed

### Model implementation

The model was implemented in R using the *rstan* package. [41] The model initializes 28 days before the first reported case or death. Given the delay from infection to death, we chose 28 days to allow the model to generate the necessary number of new infections to plausibly result in a death early in the observed time series. The model is fit to data from each county or state separately. For state-level results (including Washington, DC) we estimated outcomes using a Hamiltonian Monte Carlo algorithm. [42] The model ran for 3000 iterations (2000 burn-in) on 4 chains, and 3000 samples (across 3 chains) from the posterior were included in these results. Counties were fit using an optimization routine that reports the maximum a posteriori estimate, which represents an estimate of the mode of the posterior distribution of the model parameters.

### Covidestim package

The *covidestim* package is a package for the R programming language, suitable for public as well as research use. It can accommodate a number of data inputs. Users may enter a vector of daily case counts and/or daily death counts. These data sources can be used in combination, so long as they are the same length and cover the same time period; days with no observed events may be represented with zeroes.

The package contains default model priors for progression probabilities and delays, detection probabilities and delays, and reporting delays associated with each data type. Users have the ability to override these defaults, though we recommend that they only specify priors for reporting delays; we do not recommend that users change default priors on parameters related to the natural history of COVID-19.

### Covidestim.org and code repositories

We produce daily estimates of COVID-19 infections and the effective reproduction number of SARS-CoV-2 at the state- and county-levels at https://covidestim.org. To allow for daily production of model estimates for all U.S. counties and states, we developed several tools. The *covidestim* Docker image is a container which allows for model execution in any HPC or cloud environment, and is the easiest way to begin using the *covidestim* R package. The *covidestim-sources* repository enables automated, version-controlled, reproducible data cleaning of four different case/death data sources by leveraging Git’s submodules feature. Finally, the *dailyFlow* repository uses the Nextflow workflow engine [43] to clean the data, orchestrate 3200+ model runs within three supported execution environments (local, HPC, cloud), and export the results for research use and for web consumption. These repositories can be found at https://github.com/covidestim, and contain extensive documentation.

## Supporting information

S1 Text Derivation of Eq 1. (DOCX)

S1 Fig Model fit to data for four states–California, Florida, New York, South Dakota. (TIFF)

S2 Fig Relationship between COVID-19 infection fatality rate (IFR) and modeled outcomes, using North Dakota as an example. (TIFF)

S3 Fig Impact of spline knot width on R_t_ estimates for four states–California, Florida, New York, South Dakota. (TIFF)

S4 Fig County-level cumulative incidence estimates for December 31, 2020, comparing main analysis results to a sensitivity analysis in which county IFRs were fixed at the state average. (TIFF)

S5 Fig Comparison of major model outcomes for selected states, under alternative assumptions for the average delay between infection and development of symptoms, for individuals developing symptomatic COVID-19. (TIFF)

S6 Fig 95% Credible intervals for key parameters, across states. (TIFF)
