# Supplementary material for: Reconstructing the course of the COVID-19 epidemic over 2020 for US states and counties: Results of a Bayesian evidence synthesis model
Source: PLoS Comput Biol. 2022 Aug 30;18(8):e1010465. doi: 10.1371/journal.pcbi.1010465 (PMC9467347; doi:10.1371/journal.pcbi.1010465)
Supplement: S1 Text — (DOCX) [file pcbi.1010465.s001.docx]

Supplementary Materials

*Derivation of Equation 1*

In Equation 1 we calculate the change in daily infections as a function of *R_t_* and the average serial interval:

*A_t+z_ = A_t_ R_t_*

In this equation *A_t_* is the number of infected individuals on day *t*, *R_t_* is the effective reproductive number on day *t*, and *z* is the mean serial interval. In this equation, *R_t_* quantifies the rate of exponential growth based on the formula $R^{-1}=\int_{a=0}^{\infty} e^{-ra}g(a)da$, where *r* is the growth rate and *g(a)* is the generation time density [1]. As this approach is based on the mean serial interval (rather than considering how infectiousness changes over time since infection), the *R_t_* values generated using this approach represent an approximation.

If *R_t_* is fixed over the days of interest, then *A_t_* will increase or decline log-linearly:

*(A_t+z_/A_t_) = (A_t+1_/A_t_)^z^*

We can now solve for *A_t+1_* as a function of *A_t_* and *R_t_*:

*R_t_ = A_t+z_/A_t_*

*R_t_ = (A_t+1_/A_t_)^z^*

*R_t_^1/z^ = A_t+1_/A_t_*

*A_t+1_ = A_t_ R_t_^1/z^* (Equation 1)

In this formulation, changes in *R_t_* directly affect the number of new cases on day *t*, and for this reason this operationalization of *R_t_* is consistent with the instantaneous reproduction number described by Cori et al [2], as compared to a Wallinga and Teunis–type case reproduction number [3].

References

[1] Wallinga J, Lipsitch M. How generation intervals shape the relationship between growth rates and reproductive numbers. *Proc Biol Sci* 2007;274(1609):599-604.

[2] Cori A, Ferguson NM, Fraser C, Cauchemez S. A new framework and software to estimate time-varying reproduction numbers during epidemics. *Am J Epidemiol* 2013;178(9):1505-12.

[3] Wallinga J, Teunis P, Different epidemic curves for severe acute respiratory syndrome reveal similar impacts of control measures. *Am J Epidemiol* 2004; 160(6):509-16.
