# Supplementary figures and images for: Reconstructing the course of the COVID-19 epidemic over 2020 for US states and counties: Results of a Bayesian evidence synthesis model
Source: PLoS Comput Biol. 2022 Aug 30;18(8):e1010465. doi: 10.1371/journal.pcbi.1010465 (PMC9467347; doi:10.1371/journal.pcbi.1010465)

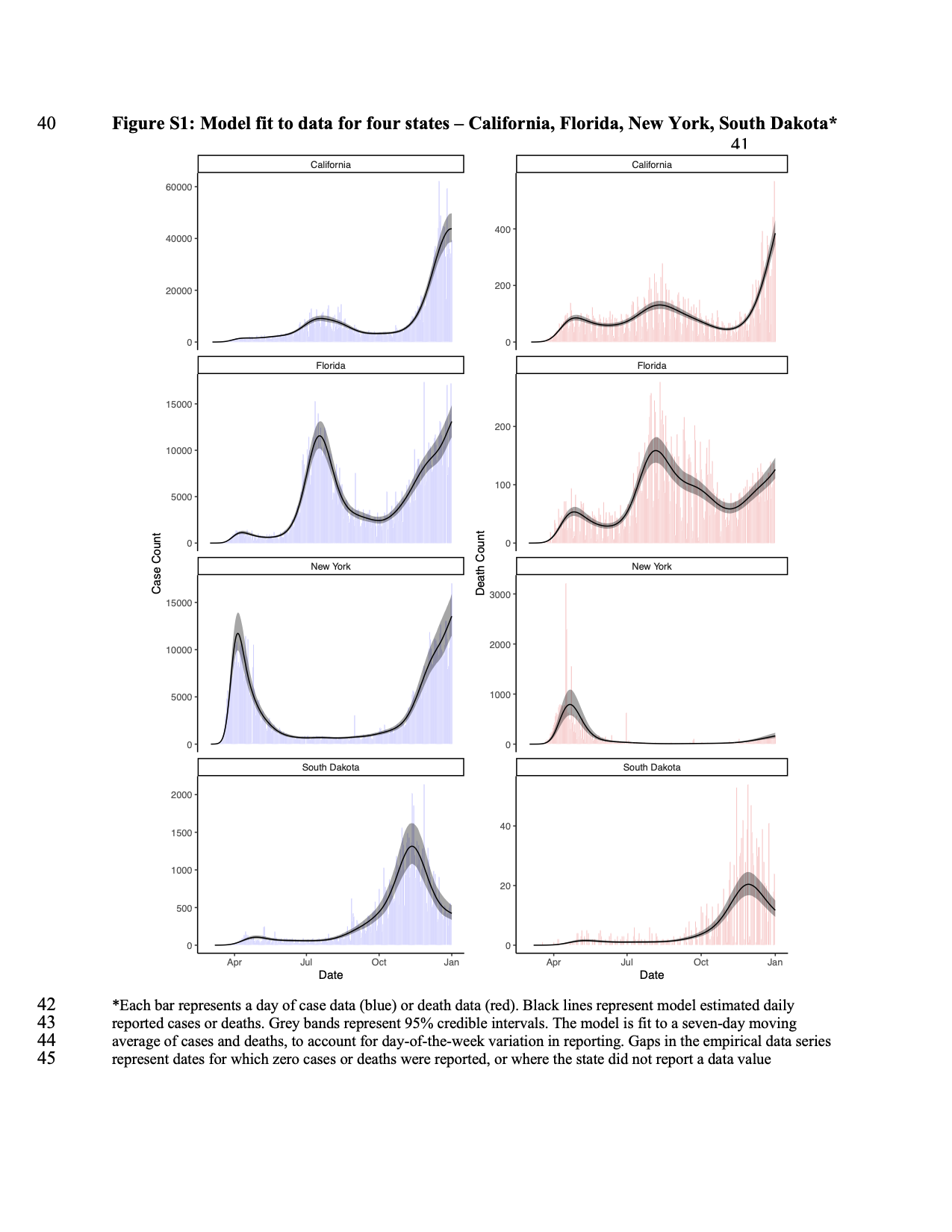

Supplement: S1 Fig — (TIFF) [file pcbi.1010465.s002.tiff]

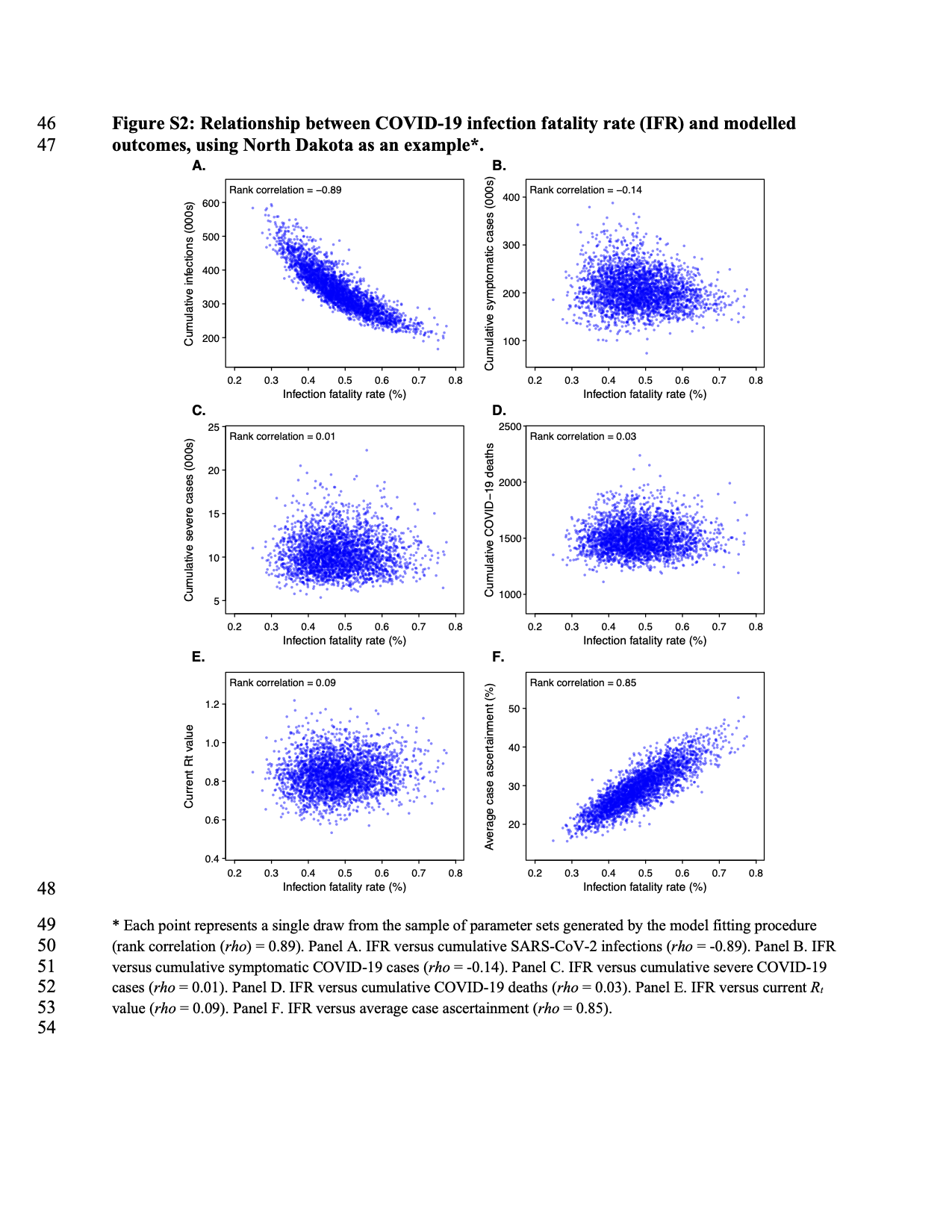

Supplement: S2 Fig — (TIFF) [file pcbi.1010465.s003.tiff]

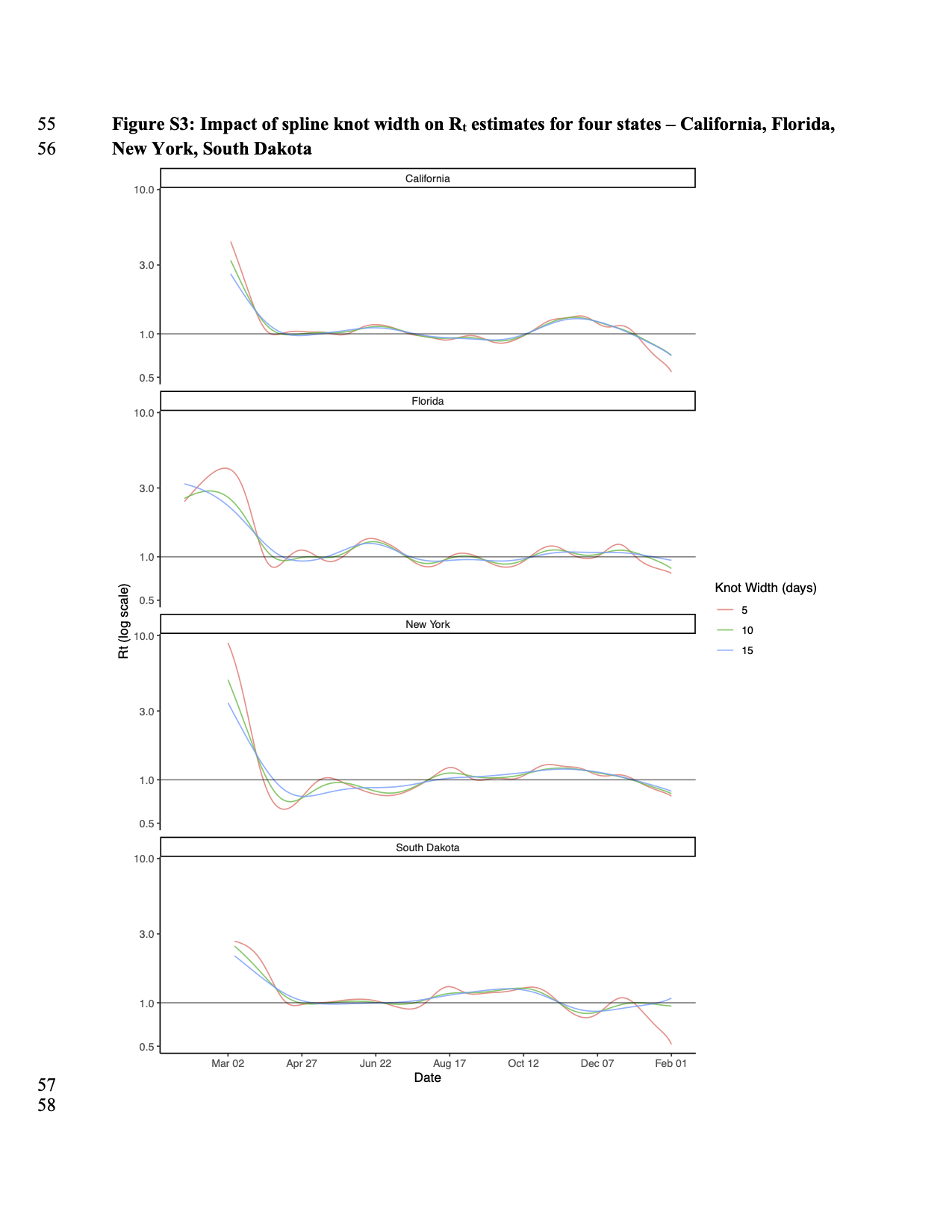

Supplement: S3 Fig — (TIFF) [file pcbi.1010465.s004.tiff]

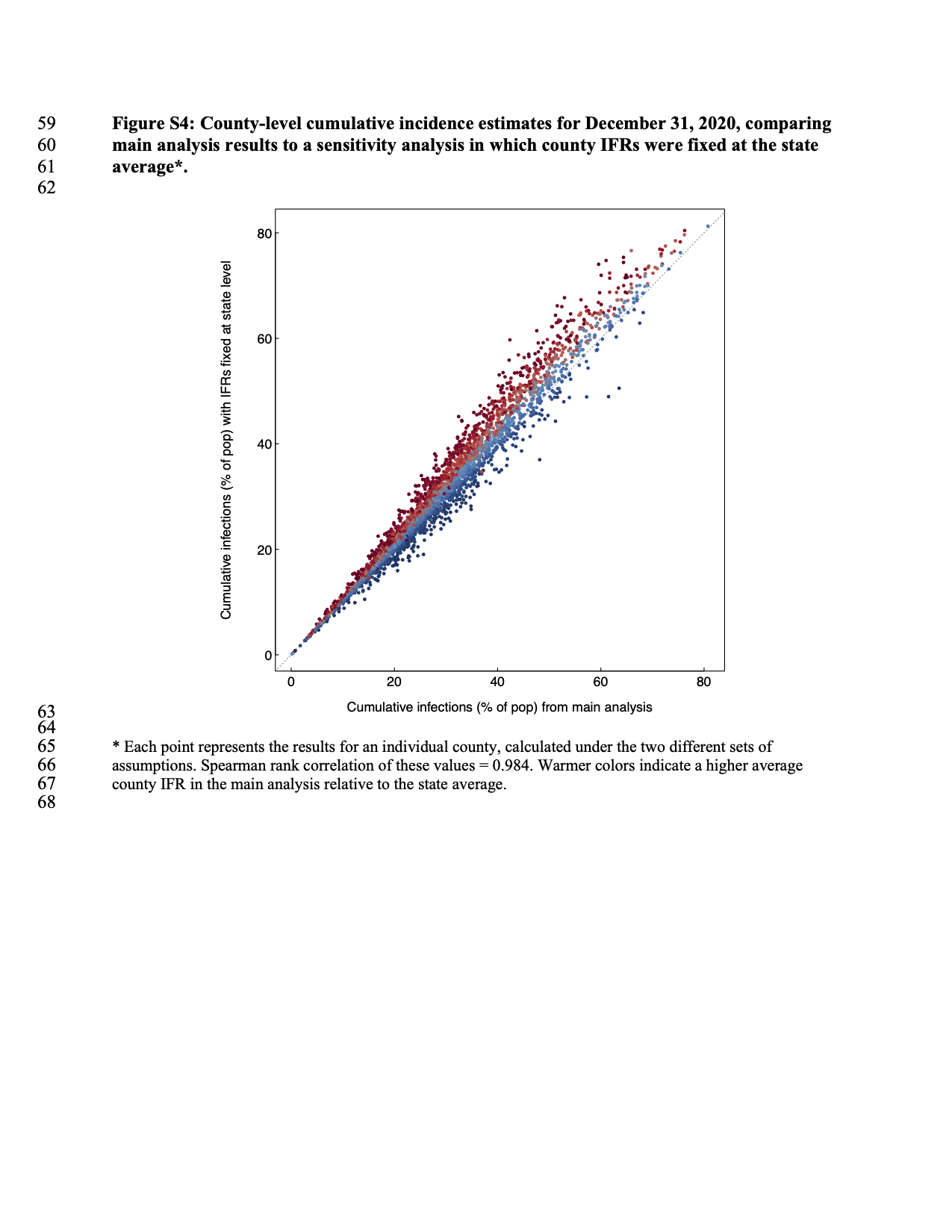

Supplement: S4 Fig — (TIFF) [file pcbi.1010465.s005.tiff]

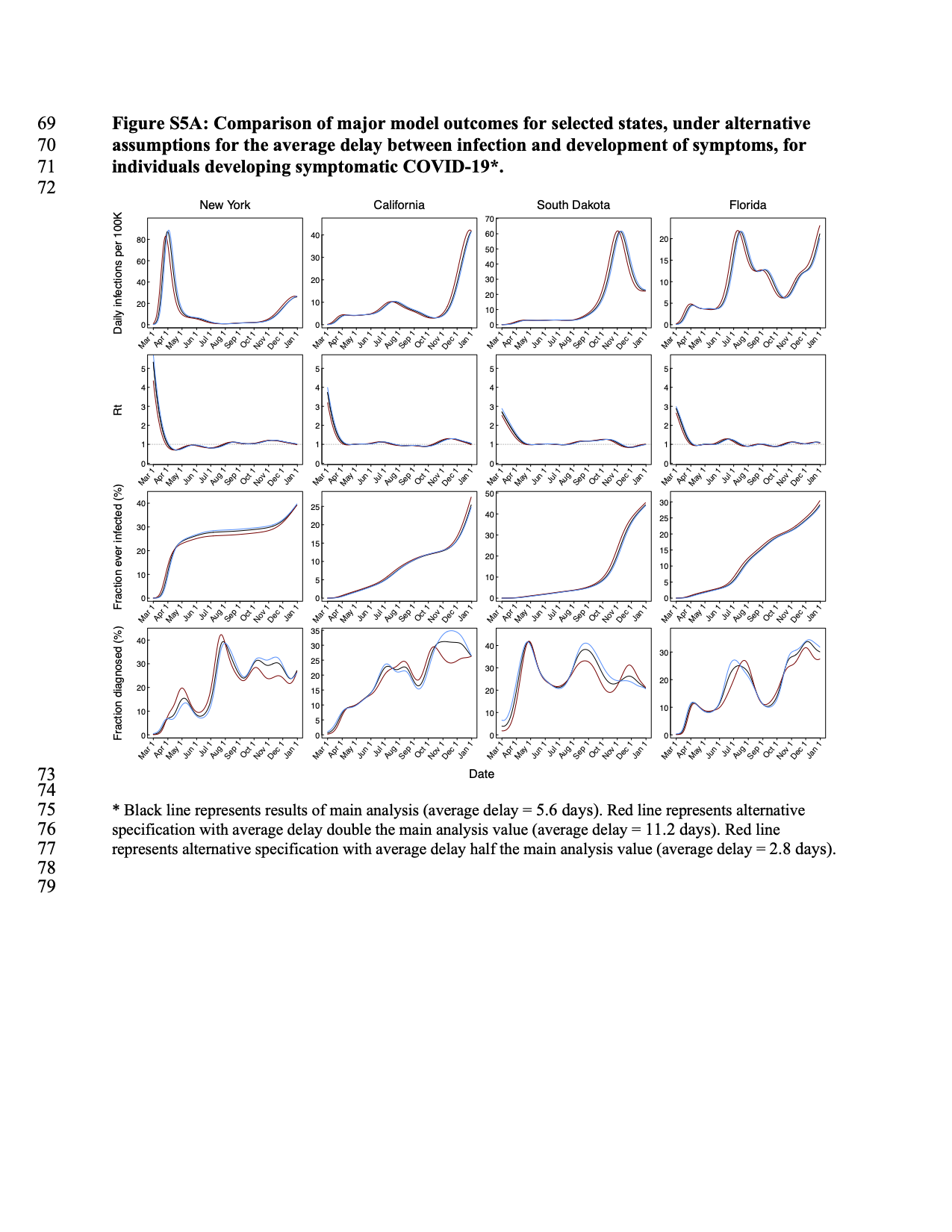

Supplement: S5 Fig — (TIFF) [file pcbi.1010465.s006.tiff]

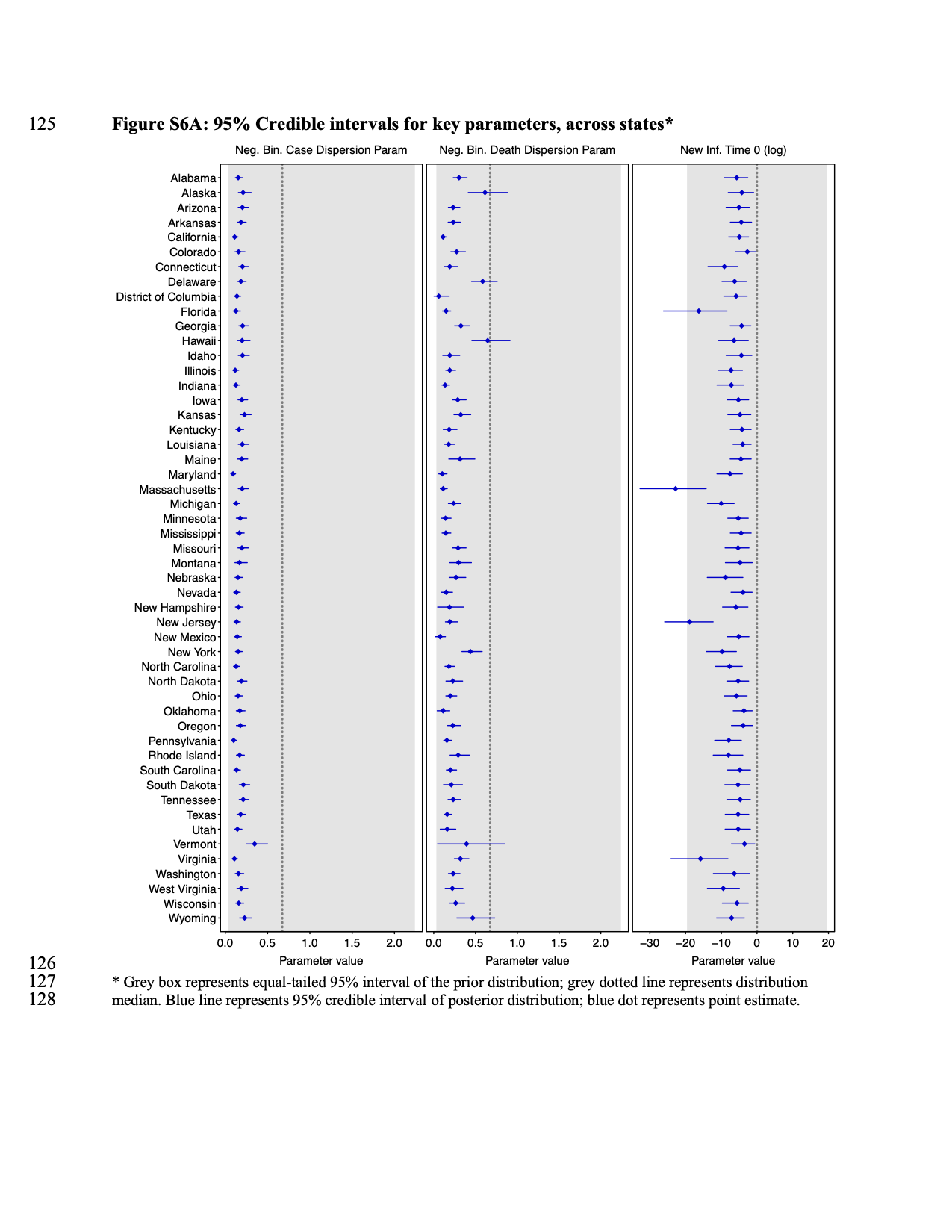

Supplement: S6 Fig — (TIFF) [file pcbi.1010465.s007.tiff]
